# Burdens of *Ascaris* spp. and *Cryptosporidium* spp. parasites in farm pigs in Ghana

**DOI:** 10.1002/vms3.756

**Published:** 2022-02-01

**Authors:** John Asiedu Larbi, Seth Offei Addo, George Ofosu‐Amoako, Uduakobong Christopher Offong, Efua Maclean Odurah, Samuel Kuranchie Akompong

**Affiliations:** ^1^ Department of Theoretical and Applied Biology College of Science KNUST, PMB Kumasi Ghana; ^2^ Parasitology Department Noguchi Memorial Institute for Medical Research University of Ghana, Legon Accra Ghana

**Keywords:** *Ascaris*, *Cryptosporidium*, Ejisu‐Juaben, pigs, prevalence

## Abstract

**Background:**

Worldwide, intestinal parasites significantly affect the health and production of pigs.

**Objective:**

This study assessed the prevalence of *Ascaris* and *Cryptosporidium* infection in pigs in the Ejisu‐Juaben Municipality of Ghana.

**Method:**

Faecal samples from two hundred (200) pigs on four different farms (labelled A, B, C, D) were processed using the Kinyoun modified Ziehl‐Neelsen method for *Cryptosporidium* and the Formol‐ether sedimentation method for *Ascaris* and microscopically examined to identify parasites to the genus level.

**Results:**

The prevalence of *Ascaris* and *Cryptosporidium* in the pigs was 76% and 77%, respectively. The weaners had the highest *Ascaris* prevalence (96.15%) with the piglets recording the least (59.25%). On the other hand, the piglets had the highest prevalence (88.89%) for *Cryptosporidium* with the boars, sows and weaners recording 75.86%, 75.42% and 73.08% respectively. The prevalence of *Ascaris* was high in farm D (78.57%) while *Cryptosporidium* was highest in farm C (86.11%). Generally, there was a significant difference (*p* = 0.044) in the mean distribution of *Cryptosporidium* in the pigs.

**Conclusion:**

The high burden of *Ascaris* and *Cryptosporidium* infections in the pigs suggest the need to adopt and implement effective control measures.

## Graphical abstract

A high burden of Ascaris and Cryptosporidium infections were observed in the sampled pigs. There is a need to employ the use of effective control measures to reduce the spread and risk of infections.

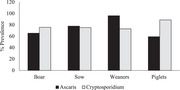

## 1 INTRODUCTION

Poor environmental hygiene leading to increased contamination of soil, water and food is reported as risk factors of parasitic infection in pigs (Levy et al., 2009). Infection of pigs with gastrointestinal parasites is also widely reported from all corners of the world and shown to be influenced by the type of pig management practised (Geresu et al., 2015). Some intestinal parasites, such as *Cryptosporidium* spp. and *Ascaris* spp., can be transmitted to other animals even including humans in many parts of the world especially in children and people with immunodeficiency diseases (Junhui et al., 2015). Although parasitic infections are usually subclinical, there are some cases of clinical infections occurring especially in growing pigs (Joachim et al., 2001; Weng et al., 2005). These parasites can restrict the growth of pigs, affect sow productivity and increase the cost of production (Knecht et al., 2011; Pedersen et al., 2002; Symeonidou et al., 2020).

Gastrointestinal parasites greatly influence the productivity of pigs and other livestock industries by causing substantial economic loss (Boes et al., 2000). Generally, the parasites are transmitted via an oral‐faecal route through infected food and water. The humid and warm conditions of the tropics as well as the deficient treatment of local pigs against parasitic diseases (Mashatise et al., 2005) invariably cause them to carry heavy burdens of gastrointestinal parasites. It is evident through various research findings of the widespread especially in Sub‐Saharan Africa, gastrointestinal parasitic infections in pigs (Nwafor et al., 2019; Omoruyi & Agbinone, 2020; Youssao et al., 2006).

To date, about 37 *Cryptosporidium* species have been recognized (Čondlová et al., 2018; Kváč et al., 2018; Ryan et al., 2014; Zahedi et al., 2017), from which *C. parvum*, *C. scrofarum*, *C. suis*, *C. tyzzeri*, *C. muris*, and *C. andersoni* have been isolated from pigs (Kváč et al., 2013; Yui et al., 2014). *Cryptosporidium parvum* is a common intestinal parasite of humans and livestock (Guselle et al., 2003). Some studies have identified *C. scrofarum* and *C. suis* infections in immunocompromised patients suggesting their zoonotic potential (Bodager et al., 2015; Cama et al., 2007; Kváč et al., 2009; Leoni et al., 2006; Wang et al., 2013; Xiao et al., 2002). However, more studies are required to determine the zoonotic potential of these Cryptosporidium species.

In the case of *Ascaris*, human infections are known to be caused by *Ascaris lumbricoides* (Ali et al., 2020) while infections in pigs are caused by *Ascaris suum* (Zheng et al., 2020). However, the interaction between humans and pigs has resulted in cross‐species transmission (Anderson, 1995; Monteiro et al., 2019; Sadaow et al., 2018) with interbreeding between *Ascaris lumbricoides* and *Ascaris suum* (Criscione et al., 2007; Peng & Criscione, 2012). More studies are required to establish the zoonotic transmission of *Ascaris* as it is unclear if pigs are significant reservoirs of human infection (Da Silva Alves et al., 2016; Leles et al., 2012).

The Ejisu‐Juaben Municipality is among the largest pig‐breeding sites in the Ashanti Region. It is a place where pigs are sold in large commercial numbers for people in Ejisu‐Juaben Municipality and even extends to reach people outside the Municipality. However, there is little to no information on the prevalence of *Ascaris* and *Cryptosporidium* among pigs in the Region. Thus, this study aimed at determining the prevalence of these intestinal parasites and further suggests the need to create and implement control measures.

## 2 METHODOLOGY

### 2.1 Sample collection

The cross‐sectional study was conducted in four intensive pig farms labelled as A, B, C and D in the Ejisu‐Juaben Municipality. The pig farms were conveniently selected based on farm size (between 50 and 80 pigs per farm) and accessibility for faecal sampling. An intensive management farming system was seen among all the farms visited.

Fresh faecal samples were collected aseptically from the pigs into separate sterile zip‐lock bags, labelled, preserved in 10% formalin and then carried to the laboratory for morphological examination of the parasites. Pigs under the age of 3 months were grouped as piglets, those within 3–6 months old were grouped as weaners and pigs 7 months and above were grouped as adult boars and adult sows. A total of two hundred (200) samples from individual pigs in four farms were collected and grouped as samples from piglets (*n* = 27), weaners (*n* = 26), adult boars (*n* = 29) and adult sows (*n* = 118).

### 2.2 Macroscopic and microscopic examination

Stool samples were examined by direct observation for mucus, blood, consistency (formed, soft or loose) and any adult parasites.

Examination of stool samples for *Ascaris* was done using the Formol‐ether concentration technique (Cheesbrough, 2006). To 2 g of a stool sample, 10 ml of formalin was added; mixture stirred using an applicator stick until a slightly cloudy suspension was attained and 4 ml of ethyl acetate added to the suspension and mixed properly for 1 min. The faecal suspension was sieved into a centrifuge tube and centrifuged for 1 min at 3000 rpm. The debris plug was loosed with an applicator stick and the supernatant decanted. One drop of the sample was placed on a clean microscope slide without any gross fibres and particles. Immediately, 1 drop of Lugol's iodine was then added and covered with a coverslip. The specimen was then examined with the low power objective lens (10×) beginning at one corner of the smear and systematically examined successive adjacent swaths with the high‐power objective lens (40×) for the eggs of *Ascaris*.

The Kinyoun modified Ziehl‐Neelsen method was used in the preparation of samples for identification of *Cryptosporidium* (El‐Moamly & El‐Sweify, 2012). Briefly, a few drops (one to two) of the specimen were then smeared on the slide and allowed to air dry. It was then fixed with absolute methanol for 1 min and dried at room temperature. Set up was flooded with Kinyoun's carbol fuchsin for 5 min. The slide was rinsed briefly (3–5 s) with 50% ethanol and thoroughly with water thereafter. The smear was decolourized with 1% sulphuric acid for 2 min or until no more colour ran from the slide. The slide was rinsed with water, drained, counterstained with methylene blue for 1 min and rinsed with water. The slide was allowed to stand for air‐drying. The prepared slide was finally mounted under the oil immersion objective for the morphological examination of *Cryptosporidium* oocyst.

### 2.3 Statistical analysis

Results were organized using Microsoft Excel spreadsheet 2010 and analysed for significant differences between the mean distribution of egg/cyst per gram of stool (*Ascaris* and *Cryptosporidium* respectively) in pigs across the farms using ANOVA with SPSS IBMS 2.0v. The prevalence was calculated for all data sets as the number of infected individuals divided by the total number of individuals examined, multiplied by 100. The level of significance was fixed at 95%.

## 3 RESULTS

**FIGURE 1:**
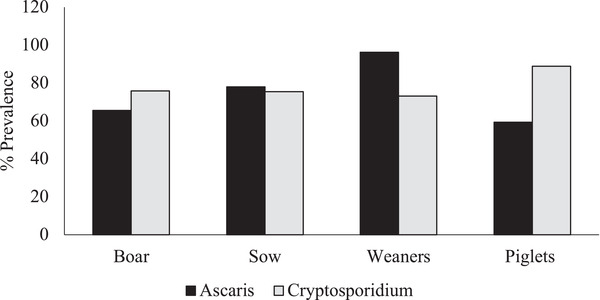
Prevalence of *Cryptosporidium* and *Ascaris* across pig groups in the farm

Out of the 200 pig samples examined, an overall prevalence of 77% of *Cryptosporidium* infection and 76% of *Ascaris* infection in pigs were recorded. (Table 1). A significant difference was observed in the mean distribution of *Cryptosporidium* in the pigs (*p* = 0.044). It was observed that boars and piglets were mostly infected with *Cryptosporidium* whereas sows and weaners were mostly infected with *Ascaris* (Figure 1).

**TABLE 1:** Overall distribution of *Cryptosporidium* and *Ascaris* across the farm

		*Ascaris* spp.		*Cryptosporidium* spp.	
Farms	Number examined	No. infected	Prevalence (%)	No. infected	Prevalence (%)
A	109	84	77.06	80	73.39
B	41	31	75.61	31	75.61
C	36	26	72.22	31	86.11
D	14	11	78.57	12	85.71
**Total**	**200**	**152**	** 76.00**	**154**	**77.00 **

### 3.1 Prevalence of C*ryptosporidium* spp. and *Ascaris* spp. across various pigs in farms

**FIGURE 2:**
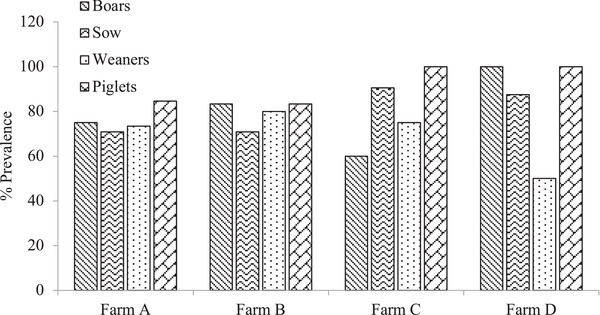
Prevalence of *Cryptosporidium* spp. across various pig groups in the farm

**FIGURE 3:**
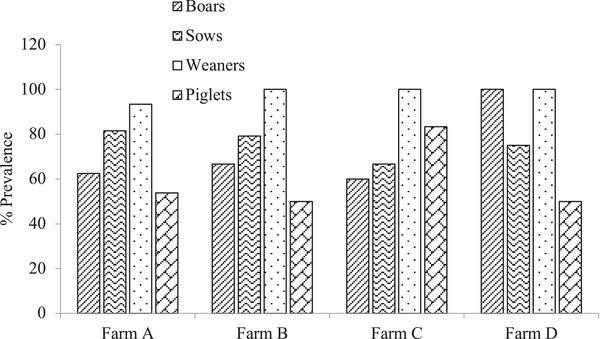
Prevalence of *Ascaris* spp. across various pig groups in the farm

The prevalence of *Cryptosporidium* spp. and *Ascaris* spp. were recorded categorically in boars, sows, weaners and piglets (Figures 2 and 3). Prevalence within the various farms showed no significant difference (*p* > 0.05).

### 3.2 Mean distribution of cyst/eggs of *Cryptosporidium* spp. and *Ascaris* spp. in pigs across the farms

The mean distribution of *Cryptosporidium* spp. in the various pig groups saw the highest infection in the boars of farm D, sows of farm C, weaners of farm A and piglets of farm A (Table 2).

**TABLE 2:** Mean distribution of *Cryptosporidium* spp. among pigs in various farm

	***Cryptosporidium* spp**.			
	Farm A	Farm B	Farm C	Farm D
Boars	3.44	4.33	2.2	6
Sows	2.58	2.92	5.05	3.75
Weaners	4.13	3.4	3.25	2
Piglets	3.38	3.33	5.67	4.5

The mean distribution of *Ascaris* spp. in the various pig groups saw the highest infection in the boars of farm D, sows of farm A, weaners of farm B and piglets of farm C (Table 3).

**TABLE 3:** Mean distribution of *Ascaris* spp. among pigs in various farm

	***Ascaris* spp**.			
	Farm A	Farm B	Farm C	Farm D
Boars	2.38	2.67	2.2	3
Sows	3.8	3.08	2.19	1.63
Weaners	3.53	5	4.25	3.5
Piglets	2.31	1.67	3.5	1.5

## 4 DISCUSSION

Gastrointestinal parasites cause significant problems in pig farming by affecting pig health, increasing morbidity in younger animals and extreme cases of death (Kagira et al., 2012; Pinilla et al., 2020). Studies suggest that *Ascaris* and *Cryptosporidium* infections in pigs could be of public health importance (Cavallero et al., 2013; Zhang et al., 2013); thus there is a need to establish the current prevalence in pigs and the potential risk to farmers.

In Ghana, *C. hominis* and *C. parvum* have been identified in children within the Ashanti Region (Eibach et al., 2015). Furthermore, *Cryptosporidium* spp. was found to be the cause of diarrhoea in children reporting to the Korle bu Teaching Hospital in Accra (Adjei et al., 2004). Within the coastal savannah zone of Ghana, *Cryptosporidium* species were identified in farmers and livestock with *C. parvum* occurring in both humans and animals (Squire et al., 2017). It is important to note that *C parvum* is a zoonotic pathogen (Adegbola et al., 1994; Yu & Seo, 2004) hence livestock could play the role of reservoirs in the spread of infections.

In this study, even though there was no significant difference between the *Cryptosporidium* prevalence recorded from the various farms (*p* > 0.05), it was observed that piglets were mostly infected with *Cryptosporidium*. It has been established that *Cryptosporidium* oocyst can survive for longer periods in the faecal matter as compared to bacterial pathogens (Hutchison et al., 2005). Thus, a lack of efficient hygienic practices on the farm could enhance the spread of this parasite. In addition, piglets being in close confinement with the sows could facilitate the easy transmission of infection from the sow (Fablet, 2009). The use of water from a well and borehole on the farms could also influence the distribution of *Cryptosporidium* (Karanis et al., 2007). Originally isolated from pigs (Kváč et al., 2013; Yui et al., 2014), some studies have suggested the potential zoonotic transmission of *C. scrofarum* and *C. suis* to humans (Bodager et al., 2015; Wang et al., 2013; Xiao et al., 2002).

Although *Ascaris lumbricoides* infects humans (Ali et al., 2020) and *Ascaris suum* infects pigs (Zheng et al., 2020), there is evidence of cross‐species transmission between humans and pigs within the same location (Anderson, 1995; Monteiro et al., 2019; Sadaow et al., 2018). Furthermore, *Ascaris lumbricoides* and *Ascaris suum* can interbreed, posing a serious threat to public health (Criscione et al., 2007; Peng & Criscione, 2012). Although it is unclear if pigs are key reservoirs of human illnesses globally (Da Silva Alves et al., 2016; Leles et al., 2012), the possibility of *Ascaris* zoonotic transmission cannot be ruled out.

In Ghana, *Ascaris lumbricoides* has been identified in children at the hospital (Mirisho et al., 2017), school children (Orish et al., 2019), inhabitants of an orphanage (Duedu et al., 2015) and pregnant women (Abaka‐Yawson et al., 2020). Even though there is sparse information on *Ascaris* infection in livestock, studies have detected this parasite in goats, cattle and pigs (Atawalna et al., 2016; Futagbi et al., 2015; Mensah et al., 2018). In this study, the significant difference between the prevalence of *Ascaris* infection of the weaners and the boars, sows and piglets, and the sows and weaners could be due to the acquired immunity obtained by both the boars and sows over a consistent period of exposure to the source of infection.

It is important to note that the high prevalence of *Ascaris* infections in pigs is due to the large number of eggs produced and their ability to survive over a longer period (Hagel & Giusti, 2010). Poor environmental hygiene is said to also maintain or increase the intensity of *Ascaris* infection (Stothard et al., 2008). Additionally, *Cryptosporidium* oocysts have a protective wall that facilitates their survival in water and other environments (Thompson et al., 2008). This same protective coat makes the oocyst resistant to chlorination in water treatment (Bichai et al., 2008) and medical control of the oocysts in the pigs becomes difficult. Thus, to improve the health of pigs and increase production, it is necessary to adopt effective control measures against intestinal parasite infections and prevent or reduce transmission to farmers as well as exposure to the environment.

## 5 CONCLUSION

The study revealed a high prevalence of *Cryptosporidium* and *Ascaris* among pigs in the various farms. These parasites affect animal health and could potentially be transmitted to humans; thus, there is a need of establishing control measures to reduce the burden of infections. Furthermore, it is suggested that molecular studies be carried out in the pig farms to determine the specific species of intestinal parasites causing infections.

## AUTHOR CONTRIBUTIONS

Seth Offei Addo: conceptualization, data curation, investigation, methodology, project administration, supervision, writing – original draft, writing – review & editing.

## CONFLICT OF INTEREST

The authors declare that there are no conflicts of interest.

## ETHICAL STATEMENT

Voluntarily voided stools were used hence the animals were not directly handled. Verbal consent and permission was sought and obtained from farm owners prior to sample collection.

### PEER REVIEW

The peer review history for this article is available at https://publons.com/publon/10.1002/vms3.756

## Data Availability

All the data supporting the findings have been included in this article.
